# Pulp Extirpation

**Published:** 1905-06-15

**Authors:** Elgin Mawhinney


### PULP EXTIRPATION.

ELGIN MAWHINNEY, D.D.S.

I shall use my space this issue to send an afterword to the alumni regarding the use of cocain hydrochlorate as an aid to immediate pulp extirpation.

Much has been said and written lately on the subject of arsenic versus cocain for pulp removal, and it is not my intention to go into the merits or demerits of either drug, but rather to remind us all that there is the patient to be considered in these operations.

We cannot ignore the fact (however much we sometimes might like to) that we are operating on human beings capable of suffering. There is such a thing as becoming so enthusiastic over a method of operating that we disregard this important item.

There is no dental operation so much dreaded by the average person as that of "taking out a nerve," and if I can judge aright from the evidences at hand, this dread is greatly on the increase since the method of using cocain for this purpose has become so generally used. There is scarcely a day goes by but some one asks if I "use that method," or some one comes with the statement that "I understand you do not always use it." I am quite certain that at least twenty-five families have come into my hands the past year because former dentists had "half murdered" them with this so-called painless method. At the present writing I have a gentleman in my care who has let his teeth go without attention until they are almost hopelessly ruined because one of our very best operators attempted to remove the pulp from an upper molar, using the cocain method. The pain occasioned was so severe that the gentleman then and there decided to let all his teeth go until they troubled him severely and then have them all extracted.

The cocain enthusiast will say this is an exceptional case, but from my personal observation, as well as the experience of many operators, I think such cases occur altogether too frequently.

I do not think in performing operations on the teeth we are ever justified in causing such severe pain that patients will forever dread the dental chair. I, for one, prefer to take a little more time, make a few extra sittings if necessary, and avoid such results, and I find that patients appreciate and are willing to pay liberally for such treatment.

I do not wish to be understood as saying that the cocain method cannot, under certain conditions, be successfully used, but I do think it is not a method for universal application, and simply wish to remind us that there is to this question a patient's side, which, after all, is the most important.—*Northwestern Journal.*

